# A pilot and feasibility study to assess children’s consumption in quick-service restaurants using plate waste methodology

**DOI:** 10.1186/s12889-017-4171-5

**Published:** 2017-03-15

**Authors:** Juliana F. W. Cohen, Susan B. Roberts, Stephanie Anzman-Frasca, Madeleine M. G. Gamache, Vanessa M. Lynskey, Emilia Matthews, Megan P. Mueller, Shanti Sharma, Christina D. Economos

**Affiliations:** 1 Department of Health Sciences, Merrimack College, 315 Turnpike Street, North Andover, MA 01845 USA; 2 Department of Nutrition, Harvard T.H. Chan School of Public Health, Boston, MA USA; 3 Human Nutrition Research Center on Aging, Tufts University, Boston, MA USA; 4 Department of Pediatrics, University at Buffalo, Buffalo, NY USA; 5 ChildObesity180, Friedman School of Nutrition Science and Policy, Tufts University, Boston, MA USA; 6 Friedman School of Nutrition Science and Policy, Tufts University, Boston, MA USA

**Keywords:** Quick-service restaurants, Child diet, Plate-waste, Bomb calorimetry, Dietary assessment, Fast food, Feasibility

## Abstract

**Background:**

Children regularly consume foods from quick-service restaurants (QSR), but little is known about the foods that children order, the calories and nutrients consumed, the accuracy of stated calorie information, or the ability to assess food orders and consumption in QSRs. This study evaluated the feasibility of plate waste collection in QSRs and examined children’s orders and consumption of meals from the standard and children’s menus. Additional aims were to examine if the meals ordered met healthier standards for children’s menu items and determine the accuracy of the QSR-stated energy content of foods.

**Methods:**

Fifteen QSRs, two malls, and 116 eligible parents were approached to participate in the study in 2015. Among the families recruited, children’s meal orders and consumption were analyzed using plate waste methodology, and a subsample of foods was analyzed using bomb calorimetry in 2015.

**Results:**

Two individual QSRs and one mall food court with two QSRs agreed to participate, and *n* = 50 participants (parents with children between the ages of 5-10 years) were recruited. Children consumed on average 519 calories, 5.7 g saturated fat, 957 mg sodium, 3.7 g fiber, and 22.7 g sugar. Children ordered and consumed significantly fewer calories and less sodium and sugar with meals ordered exclusively from the children’s menu compared with the standard menu. Overall there were no significant differences between the measured and stated energy contents of the QSR foods.

**Conclusions:**

Conducting plate waste research in QSRs is feasible and there is concordance with stated calorie information. Consuming foods exclusively from the children’s menu may help limit overconsumption in QSRs.

**Electronic supplementary material:**

The online version of this article (doi:10.1186/s12889-017-4171-5) contains supplementary material, which is available to authorized users.

## Background

In the United States, approximately 33% of children consume food items (i.e., entrees, beverages, desserts, and sides [including condiments]; herein referred to as “foods”) from quick-service restaurants (QSRs) on any given day, making QSR foods the second-largest source of calories in children’s diets [1–4]. Some research suggests a positive association between QSR consumption and body mass index (BMI) among children and adolescents [5–8], possibly due to consumption of more calories, saturated fat, sugar, and sugar-sweetened beverages (SSBs) and fewer fruits and vegetables when QSRs foods are eaten [3, 9].

Research is needed to better understand the calorie and nutrient content of foods ordered and consumed by children in QSRs, and whether differences in consumption exist when children order from the standard versus children’s menu. An important limitation of existing studies on QSR consumption is the use of self-reported data, such as 24-hour recalls, which rely on a child’s or caregiver’s memory [2–4, 9]. It is currently unknown if collecting plate waste data, a gold standard for evaluating consumption, is feasible in QSRs. Additionally, plate waste methodology relies on nutrition information from QSRs to determine calories ordered and consumed; however, a limited number of studies have examined the accuracy of the calorie information. Previous studies have focused only on standard menus, not children’s menus, and the results have been mixed [10–12].

Given these gaps in knowledge, this pilot and feasibility study aimed to: (1) determine the feasibility of collecting plate waste in QSRs; and (2) examine children’s selection and consumption of QSR foods to evaluate calories and nutrients consumed and differences when ordering foods from the standard versus children’s menu. Secondary aims were to: (1) examine children’s selection of meals compared with nutrition standards for QSRs; and (2) evaluate the accuracy of QSR-stated energy content of a subsample of foods.

## Methods

A pilot and feasibility study was conducted by ChildObesity180 at the Friedman School of Nutrition Science and Policy at Tufts University, with data collection taking place at two large national restaurant chains. Stand-alone QSRs (*n* = 15) and malls with food courts (*n* = 2) containing the selected QSRs were approached to participate. Locations were based on proximity to the Greater Boston area, with an emphasis on socioeconomically diverse settings. Two stand-alone QSRs (both from the same QSR chain [“QSR Chain 1”]) and one mall with two QSRs in the food court (QSR Chain 1 and a second chain [“QSR Chain 2”]) agreed to participate (hereafter referred to as “QSRs”). This study was approved by the Tufts University Institutional Review Board.

### Participants

Participants were parents or legal guardians (herein referred to as “parents”) who were at least 18 years old and purchasing food for a child between the ages of 5-10 years for onsite consumption. A total of *n* = 55 parents were recruited (47% of the eligible parents approached). Written informed consent was not required, but research assistants explained all relevant study information to participants and provided them with a participant information document.

### Recruitment and plate waste methodology

Data were collected on 10 weekend days over three months (January through March 2015), between 11 am and 8 pm. All adults with a child in the restaurant were approached at one of two time points: (1) while waiting in line to order but after finalizing their meal selections (to minimize influencing meal choices); or (2) immediately after placing their order. After confirming eligibility, recruited participants provided their sales receipt. Additionally, at the end of the meal, participants provided any leftover foods from the child’s meal. If more than one child with the parent met the age criterion, the child with the closest birthday to the date of data collection was chosen to ensure random selection. At QSR locations with self-serve soda fountains (*n* = 2), the child’s drink cup was weighed after ice was placed in it and after the drink was poured. Food sharing was discouraged, but if sharing did occur, participants reported the amount shared at the end of the meal.

At the end of the meal, parents provided all remaining foods and containers/wrappers (including condiment packets) from the child’s meal. Additionally, parents described all of the foods ordered for the child, including the size and any modifications to the menu items, and completed a survey which included demographic information for both the parent and child. If a parent wanted to bring remaining foods home, leftovers were weighed on site (*n* = 12 food items). Otherwise, foods were brought back to the lab in sealed bags for weighing. Foods were weighed in grams using food scales (OXO 1130800, OXO Company).

Two samples of every food ordered by the participants were purchased to obtain pre-consumption weights (for fountain sodas, the weights of the ice and drink were replicated). Consumption was calculated using the formula:

$$ \frac{\mathrm{Average}\ \mathrm{preweight}\ \mathrm{of}\ \mathrm{the}\ \mathrm{food}\ \left[\mathrm{based}\ \mathrm{on}\ 2\ \mathrm{samples}\right]\hbox{-} \mathrm{Postweight}\ \mathrm{of}\ \mathrm{the}\ \mathrm{food}}{\mathrm{Average}\ \mathrm{preweight}\ \mathrm{of}\ \mathrm{the}\ \mathrm{food}\ \left[\mathrm{based}\ \mathrm{on}\ 2\ \mathrm{samples}\right]} \times 100 $$

For shared items, parents were asked to estimate the percent or quantify the number of an item consumed by the child (e.g., ten fries); when a number was provided, this quantity from the pre-weight sample was weighed to estimate the post-consumption weight. The percentage consumed calculated for each food item was multiplied by the stated nutrient contents available on the QSRs’ websites to determine nutrients consumed for that food item. Nutrients consumed for each food item (i.e., entrée, beverage, side, condiments, and dessert) were summed to determine the overall nutrients consumed by the child.

The nutrients from the meals were compared with the National Restaurant Association’s Kids LiveWell standards for children’s meals which require at least two healthy components (fruit, vegetable, whole grain, lean protein, and/or lower-fat dairy [1% or skim]) and include limits for calories (≤600 kcal), total fat (≤ 35% of calories) saturated fats (≤ 10% of calories), trans fat (≤ 0.5 g), sodium (≤ 770 mg), and sugar (≤ 35% of calories) [13].

### Bomb calorimetry methodology

Bomb calorimetry was used to evaluate the accuracy of the QSR-stated energy content of foods, with methods based on those previously reported [10, 11]. Twenty unique foods were randomly selected from foods ordered by participants (*n* = 10 foods from QSR Chain 1 and *n* = 10 foods QSR Chain 2) for energy content analyses using a bomb calorimeter, which is typically accurate to a mean (SD) of -1.9% (0.3%) [10]. Only entrées, side dishes, beverages, and desserts were analyzed; condiments were analyzed only if they were included as part of a meal (e.g., a hamburger with ketchup). Food samples were placed in sealed bags and frozen. Pre-consumption samples (two samples of each food combined) were analyzed to assess the accuracy of the QSR-stated energy content of the foods, and in secondary analyses, the corresponding post-consumption samples were analyzed to evaluate the accuracy of plate waste methodology in QSRs. After weighing, samples were blended to a homogenous consistency, freeze-dried (Virtis Benchmark 1000 Lyophilizer, Virtis Co, Gardiner, NY), ground into a fine powder, and pressed into pellets of approximately 1 g each. Gross energy was determined using an Isoperibol Bomb Calorimeter (Parr model 1261, Parr Instrument Co, Moline, IL), with benzoic acid (Benzoic Acid 1 g pellets, Parr Instrument Co, Moline, IL) used as the standard for calibration. Total energy content for each food sample was determined by multiplying the total weight from the dried foods by the mean heat of combustion from the samples (accounting for duplicate samples where applicable).

Because bomb calorimetry determines the gross energy content of foods, while the stated energy is a metabolizable energy estimation (i.e., the gross energy adjusted for obligatory energy losses in urine and feces), QSR-stated energy was converted to gross energy equivalents using the formula: *gross energy = (fat [in grams]) × 9.4) + (protein [in grams]) × 5.65) + (total carbohydrate [in grams]) × 4.15)* [10, 14].

Of the 20 pre-consumption samples, three were later excluded. One was determined to be a non-caloric diet beverage. Two other beverage samples were served by employees at QSRs that did not have a self-serve soda fountain and contained substantial levels of ice (but the exact quantity was unknown) while the QSR-stated energy information was for a beverage without ice. Therefore *n* = 17 pre-consumption samples were analyzed. Seven corresponding post-consumption food items were also analyzed.

### Analyses

Descriptive statistics were used to examine QSR recruitment and participant characteristics. T-tests were used to determine differences in the calories, total fat, percent of calories from total fat, saturated fat, percent of calories from saturated fat, *trans* fat, sodium, fiber, sugar, and percent of calories from sugar for the foods ordered and consumed from the standard versus children’s menu. Child age and sex were not significant confounders when examining selection or consumption and therefore were not controlled for in analyses. Comparisons of the QSR-stated energy values with those determined using bomb calorimetry techniques were conducted using paired t-tests. Analyses were performed in 2015 using SAS statistical software (version 9.4, SAS Institute Inc).

## Results

### Recruitment feasibility

Of the 15 stand-alone QSRs approached, two agreed to participate (13% participation rate); of the 2 malls approached, one agreed to participate (50% participation rate). Reasons cited by QSR/mall managers for non-participation were company policies to not participate in research (*n* = 14), concerns regarding negative portrayals of QSR foods in research (*n* = 1), and disruptions to customers (*n* = 1). Of *n* = 116 eligible parents approached, *n* = 55 were recruited (47% recruitment rate). A total of *n* = 54 adults with children who were approached were not eligible to participate (32%), the primary reason being plans to consume foods offsite (35%); other reasons included a child not meeting the age requirements (24%) or the adult not being a parent/legal guardian (11%).

Among the *n* = 55 recruited parents, *n* = 5 had children who were consuming snacks only (determined by the parent survey) and were excluded from analyses. Table 1 summarizes the characteristics of the *n* = 50 participating parents and their children who consumed a meal in the QSRs. Overall, 60% of the participating parents were female, and 72% of the parents had at least a college education. The average age of the participating parent’s child was 7.6 years (SD = 1.6; Range 5.0-10.7 years). Approximately half of the children were White (48%), 22% were multi-racial, 12% were Hispanic, 12% were Asian, and 4% were Black.

**Table 1:** Characteristics of participating parents and their children (*n* = 50 parent-child dyads)

Child Characteristics	
	Mean (range)
Age (yrs)	7.6 (5.0-10.7)
	%
Female	52
Race/ethnicity	
Asian	12
Black	4
Hispanic	12
White	48
More than one race	22
Parent Characteristics	
	%
Female	60
Parent Education	
No college degree	28
College or graduate degree	72
Frequency of eating at QSRs	
> Once a month	24
Once a month	14
A few times per year	62

The majority of participants (*n* = 30; 55%) were recruited between 12:45 pm-2 pm. Data collection was substantially more efficient in the mall food court; on average, approximately two participants were recruited per hour in the mall food court QSRs compared with only one participant recruited every other hour in the stand-alone QSRs.

### Foods ordered

Overall, the total food ordered (i.e., entrées, sides, drinks, condiments, and additional desserts if applicable) for the children had on average 708 calories, 27 g of total fat, 7.3 g of saturated fat, and 0.25 g of *trans* fat (Table 2). Total orders also contained on average 1212 mg of sodium, 4.9 g of fiber, and 37.7 g of sugar. Entrées and sides accounted for the majority of total fat, saturated fat, *trans* fat, sodium, and fiber in the meal, while beverages and desserts, such as ice cream and shakes, were the primary sources of sugar. Only 12% of children (*n* = 6) had orders that included a dessert, with half of these meals ordered from the standard (*n* = 3) menu and half ordered from the children’s menu (*n* = 3).

**Table 2:** Average nutrient information for children’s meals ^a^ *ordered* by menu type^b^ in quick-service restaurants

	Mean Calories ± SD (kcal [range])	Mean Total Fat ± SD (g [range])	Mean % of Calories from Total Fat ± SD (% [range])	Mean Saturated Fat ± SD (g [range])	Mean % of Calories from Saturated Fat ± SD (% [range])	Mean Trans Fat ± SD (g [range])	Mean Sodium ± SD (mg [range])	Mean Fiber ± SD (g [range])	Mean Sugar ± SD (g [range])	Mean % of Calories from Sugar ± SD (% [range])
All Meals (*n* = 50)										
Excluding dessert^c^	665 ± 258 (190-1270)	25.6 ± 14.4 (5.0-59.0)	33.3 ± 10.1 (9.6-52.1)	6.5 ± 3.7 (1.5-17.5)	8.7 ± 3.3 (2.9- 18.3)	0.23 ± 0.40 (0.00-2.00)	1183 ± 608 (360-3630)	4.8 ± 2.4 (1.0-10.0)	32.5 ± 22.2 (0.0-79.0)	19.3 ± 14.0 (0.0-56.2)
Including dessert^c^	708 ± 254 (300-1270)	27.0 ± 14.3 (5.0-59.0)	32.9 ± 9.4 (9.6-48.3)	7.3 ± 4.3 (1.5-19.5)	9.1 ± 3.7 (2.9-18.3)	0.25 ± 0.41 (0.00-2.00)	1212 ± 603 (385-3630)	4.9 ± 2.4 (1.0-10.0)	37.7 ± 23.6 (0.0-90.0)	21.8 ± 13.5 (0.0-56.2)
Meals from the Children’s Menu (*n* = 18)										
Excluding dessert^c^	553* ± 182 (190-885)	22.6 ± 8.5 (11.0-38.3)	36.8* ± 6.0 (24.6-52.1)	5.5 ± 2.6 (2.0-12.0)	8.8 ± 2.3 (5.5-12.2)	0.21 ± 0.28 (0.00-0.67)	876* ± 323 (360- 1575)	3.8* ± 1.6 (2.0-7.0)	25.3* ± 12.2 (0-42.3)	17.5 ± 9.5 (0-35.2)
Including dessert^c^	633 ± 232 (300-1140)	25.0 ± 10.1 (11.0-46.0)	35.1 ± 4.2 (24.6-42.6)	7.1 ± 4.5 (2.0-19.5)	9.6 ± 3.2 (5.5- 16.6)	0.27 ± 0.33 (0.00-0.67)	929* ± 362 (385-1925)	3.9* ± 1.5 (2.0-7.0)	35.8 ± 22.9 (6.0-90.0)	22.0 ± 9.5 (5.7-40.0)
Meals from the Standard Menu (*n* = 32)										
Excluding dessert^c^	728 ± 275 (310-1270)	27.2 ± 16.7 (5.0-59.0)	31.3 ± 11.4 (9.6-48.3)	7.0 ± 4.1 (1.5-17.5)	8.6 ± 3.8 (2.9-18.3)	0.23 ± 0.46 (0-2.00)	1355 ± 665 (470-3630)	5.4 ± 2.6 (1.0-10.0)	36.5 ± 25.5 (0-79.0)	20.4 ± 16 (0-56.2)
Including dessert^c^	750 ± 260 (310-1270)	28.1 ± 16.2 (5.0-59.0)	31.6 ± 11.2 (9.6-48.3)	7.5 ± 4.3 (1.5-17.5)	8.8 ± 4.0 (2.9-18.3)	0.23 ± 0.46 (0-2.00)	1371 ± 655 (470-3630)	5.5 ± 2.6 (1.0-10.0)	38.8 ± 24.3 (0.0-79.0)	21.7 ± 15.4 (0.0-56.2)

^*^ Significantly different (*p* < 0.05) compared with meals ordered from the standard menu (e.g., a meal from the children’s menu excluding dessert compared with a meal from the standard menu excluding dessert), using t-tests

^a^ Nutrition information for the meals include the entrée, beverage, and side dishes combined

^b^ Menu type refers to meals ordered from a children’s menu or a standard menu (a.k.a. “adult” or “core” menu)

^c^ Desserts included cookies, ice cream, and shakes; *n* = 6 meals included dessert (*n* = 3 with meals from the children’s menu and *n* = 3 with meals from the standard menu)

The majority of meals (*n* = 32; 64%) were from the standard menu; there were no significant differences in the likelihood of selecting from the standard versus the children’s menu based on the child’s age. Desserts were not part of the children’s menus, but were sometimes ordered in addition to a meal. Meals ordered exclusively from the children’s menu (excluding dessert) contained significantly fewer calories, total fat, sodium, sugar, and fiber compared with meals ordered from the standard menu without dessert. When dessert was added to a children’s menu order, calories, total fat, and sugar were similar to meals ordered from the standard menu with a dessert; sodium and fiber remained significantly lower. Two out of 50 meals ordered (both from the children’s menu) met the nutrition standards against which meals were compared; this represented 11% of orders from the children’s menu and 4% of total orders.

### Foods consumed

On average 84.2% (SD 20.5) of entrées, 66.8% (SD 34.4) of beverages, 65.1% (SD 32.2) of sides, and 88.7% (SD 27.3) of desserts were consumed. This translated to children consuming on average 519 calories, 20.2 g of total fat, 5.7 g of saturated fat, and 0.2 g *trans* fat (Table 3). Additionally, children consumed on average 957 mg of sodium, 3.7 g of fiber, and 22.7 g of sugar. Nearly a quarter of the children (24%) consumed over 600 calories, the maximum level for the Kids LiveWell program standards (Additional file 1: Figure S1). There were no significant differences by age in nutrients consumed. Compared with the standard menu, children with meals from the children’s menu consumed significantly fewer calories and less sodium and fiber when desserts were excluded (and still less sodium and fiber when including desserts).

**Table 3:** Average nutrient information for children’s meals ^a^ *consumed* by menu type^b^ in quick-service restaurants

	Mean Calories ± SD (kcal [range])	Mean Total Fat ± SD (g [range])	Mean % of Calories from Total Fat ± SD (% [range])	Mean Saturated Fat ± SD (g [range])	Mean % of Calories from Saturated Fat ± SD (% [range])	Mean Trans Fat ± SD (g [range])	Mean Sodium ± SD (mg [range])	Mean Fiber ± SD (g [range])	Mean Sugar ± SD (g [range])	Mean % of Calories from Sugar ± SD (% [range])
All Meals (*n* = 50)										
Excluding dessert^c^	486 ± 197 (152-1044)	19.1 ± 11.5 (5.0-51.0)	33.9 ± 9.86 (9.6- 52.1)	5.0 ± 3.0 (1.5-16.1)	9.2 ± 3.3 (2.9-16.2)	0.19 ± 0.36 (0.00-2.00)	935 ± 462 (273-2218)	3.7 ± 2.1 (0.7-8.0)	18.8 ± 13.0 (0.0-50.0)	16.6 ± 11.4 (0.0-42.6)
Including dessert^c^	519 ± 222 (152-1086)	20.2 ± 12.0 (5.0-51.0)	33.6 ± 9.2 (9.6- 50.0)	5.7 ± 3.8 (1.5-18.9)	9.5 ± 3.6 (2.9-16.2)	0.20 ± 0.37 (0.00-2.00)	957 ± 471 (273-2218)	3.7 ± 2.1 (0.7-8.0)	22.7 ± 17.7 (0.0-89.3)	18.4 ± 11.2 (0.0-42.6)
Meals from the Children’s Menu (*n* = 18)										
Excluding dessert^c^	414* ± 167 (152-757)	16.8 ± 6.9 (5.5-31.9)	37.0* ± 6.1 (24.4-52.1)	4.3 ± 2.4 (1.6-10.3)	9.2 ± 2.6 (5.5-14.5)	0.19 ± 0.26 (0.00-0.67)	684* ± 347 (273-1483)	2.8* ± 1.5 (0.7-7.0)	19.3 ± 11.9 (0.0-39.0)	18.0 ± 9.6 (0.0- 36.0)
Including dessert^c^	472 ± 253 (152-1086)	18.6 ± 9.8 (5.5-43.7)	35.6 ± 4.4 (24.4-43.3)	5.5 ± 4.3 (1.6-18.9)	9.8 ± 3.1 (5.5-15.6)	0.23 ± 0.31 (0.00-0.95)	723* ± 409 (273-1833)	2.9* ± 1.5 (0.7-7.0)	26.9 ± 22.6 (2.2-89.3)	21.2 ± 9.1 (5.7-36.0)
Meals from the Standard Menu (*n* = 32)										
Excluding dessert^c^	527 ± 203 (224-1044)	20.4 ± 13.3 (5.0-51.0)	32.1 ± 11.2 (9.6-50.0)	5.5 ± 3.3 (1.5-16.1)	9.1 ± 3.6 (2.9-16.2)	0.19 ± 0.41 (0.00-2.00)	1076 ± 463 (288-2218)	4.2 ± 2.2 (0.9-8.0)	18.6 ± 13.7 (0.00-50.0)	15.9 ± 12.3 (0.00-42.6)
Including dessert^c^	545 ± 202 (282-1044)	21.1 ± 13.1 (5.0-51.0)	32.5 ± 10.9 (9.6-50.0)	5.9 ± 3.5 (1.5-16.1)	9.4 ± 3.9 (2.9-16.2)	0.19 ± 0.41 (0.00-2.00)	1075 ± 462 (288-2218)	4.2 ± 2.2 (0.9-8.0)	20.3 ± 14.2 (0.00-52.7)	16.8 ± 12.1 (0.00-42.6)

^*^ Significantly different (*p* < 0.05) compared with meals ordered from the standard menu (e.g., a meal from the children’s menu excluding dessert compared with a meal from the standard menu excluding dessert), using t-tests

^a^ Nutrition information for the meals include the entrée, beverage, and side dishes combined

^b^ Menu type refers to meals ordered from a children’s menu or a standard menu (a.k.a. “adult” or “core” menu)

^c^ Desserts included cookies, ice cream, and shakes; *n* = 6 meals included dessert (*n* = 3 with meals from the children’s menu and *n* = 3 with meals from the standard menu)

### Bomb calorimetry

Table 4 presents the measured energy calculated using bomb calorimetry compared with the QSR-stated energy for *n* = 17 pre-consumption food items. In the overall sample, there were no significant differences between the stated and measured energy contents of the foods. Only two foods (12%) contained over 100 kcal/portion more than the stated energy contents.

**Table 4:** Measured versus stated energy content among a subsample of foods^a^ from quick-service restaurants (QSRs) assessed using bomb calorimetry

	Sample size	Stated Energy Mean ± SD (kcal/portion)	Measured Energy Mean ± SD (kcal/portion)	Energy Difference Mean^b^ ± SD (%^c^ ± SD) [kcal/portion]
Overall	17	409 ± 196	382 ± 162	−27 ± 59 (8.7 ± 3.3)
QSR Chain 1	9	438 ± 100	389 ± 73	−49* ± 41 (10.9 ± 2.6)
QSR Chain 2	8	374 ± 232	376 ± 271	2 ± 68 (6.1 ± 1.9)

^*^Stated Energy and Measured Energy are significantly different (*p* < 0.05) using paired t-tests

^a^ Based on the pre-consumption food samples

^b^ Calculated as Measured – Stated Energy

^c^ Calculated as the absolute percent difference in calories between the Stated Energy and Measured Energy

In QSR Chain 1, eight out of nine foods (89%) had measured energy contents at least 10 kcal/portion lower than the stated energy contents, and one food (11%) had a negligible difference between the measured and stated energy content (a difference within ±10 kcal/portion). The measured energy contents averaged 49 kcal/portion less than the stated energy contents (389 kcal/portion vs. 438 kcal/portion, respectively; *p* = 0.007) in QSR Chain 1.

In QSR Chain 2, three out of eight foods (38%) had measured energy contents higher than the stated energy content (at least 10 kcal/portion higher than stated), while three foods (38%) had measured energy contents lower than the stated energy contents (at least 10 kcal/portion lower than stated). Two foods (25%) had negligible differences between the measured and stated energy contents (a difference within ±10 kcal/portion). The average difference between the measured and stated energy content was 2 kcal/portion (376 kcal/portion measured vs. 374 kcal/portion stated; *p* = 0.95).

Calorie consumption calculated from plate waste measurements was compared with the percent consumed assessed using bomb calorimetry among a subsample of post-consumption foods that were not fully consumed (*n* = 7). There were no substantial differences between the two measures (differences were less than 10%).

## Discussion

This pilot study showed that collecting plate waste measurements in QSRs was feasible.

Overall, the majority of children ordered meals from the standard menu rather than the children’s menu, and few of these meals met designated nutrition standards. Compared with the standard menu, meals ordered from the children’s menu had significantly fewer calories, and less sodium, sugar, and fiber; children also consumed significantly fewer calories and less sodium and fiber with children’s menu meals. Calorie differences were seen only when desserts were excluded. Almost a quarter of the children consumed over 600 calories (range = 151-1086 kcal).

The primary challenge for collecting plate waste data was recruitment of QSRs. Additionally, collecting plate waste in mall food courts was more efficient than in individual QSRs due to greater numbers of eligible participants consuming foods on site and the ability to simultaneously recruit participants at different QSRs.

Children consumed on average 519 calories in QSRs. A previous study examining self-reported data from NHANES estimated that children consumed on average 576 kcal in QSRs [4]. The roughly 60 calories difference may be due to greater caloric content of the meals consumed in QSRs not included in this pilot study, errors from estimating consumption in QSRs using 24-hour recalls, or from the larger, nationally representative nature of that study’s participants. The present study also found that children consumed over 950 mg of sodium on average, roughly 40% of the recommended limit of 2,300 mg/day [15]. Additionally, children consumed 23 g of sugar on average, which is near the daily limit of 25 g/day of added sugar recommended by the American Heart Association (and nearly half of the recommended 50 g/day limit in the current Dietary Guidelines for Americans) [16, 17].

Overall there were no significant differences between the stated and measured energy contents of the foods examined. There was some variability in the accuracy of the stated energy contents of individual sampled foods, but only two (12%) of the tested foods contained a difference greater than 100 kcal/portion between the measured and stated energy contents. This suggests that it is reasonable to rely on stated calorie information when conducting plate waste studies given the high degree of standardization in QSRs. These findings are in line with a previous study examining a broad sample of standard menu QSR foods [11].

### Limitations

Because this was a pilot and feasibility study, only two QSR chains (in four locations) were examined. Additionally, QSR foods taken home for consumption were not assessed. However, previous research has found no significant differences in consumption of QSR foods in restaurants compared with at home [4]. While the participating QSRs were located in socio-economically diverse locations, participating parents were on average more highly educated than the general population. Future studies should examine differences in QSR food selection and consumption by participant socio-economic status. It is also possible that participation in this study influenced consumption. However, because research assistants discussed the study only with the parents and did not directly state that consumption would be assessed, this likely reduced any influence on the child’s consumption. Another limitation was the small number of foods analyzed using bomb calorimetry. Future studies should examine additional foods from children’s menus to confirm these results. An important strength of this study was the use of plate waste methodology, rather than self-report, to determine consumption. Additionally, this pilot study demonstrated that plate waste assessment in QSRs is feasible; future, larger studies should consider using this method to examine the role that restaurants play in children’s diets and examine the potential contribution these settings could make to reducing overconsumption and obesity in children.

## Conclusions

To our knowledge, this is the first study to examine children’s meal consumption in QSRs using plate waste methodology. While conducting research in QSRs is challenging, this study found that measured plate waste is a feasible method for assessing children’s consumption in QSRs and that stated nutrition information was reliable. This study also found that ordering from the children’s menu was associated with orders and consumption that were significantly lower in calories, sodium, and sugar, compared with orders from the standard (adult) menu, if desserts were not added to the order. Public health efforts should focus on encouraging ordering from the children’s menu among QSR patrons and infrequent dessert selections to limit overconsumption.

## Additional file

Additional file 1: Figure S1. Children’s energy consumption in quick-service restaurants. (DOCX 18 kb)

## Abbreviations

BMI: Body mass index
QSR: Quick-service restaurants
SSB: Sugar-sweetened beverage
