# Supplementary material for: A pilot and feasibility study to assess children’s consumption in quick-service restaurants using plate waste methodology
Source: BMC Public Health. 2017 Mar 15;17:259. doi: 10.1186/s12889-017-4171-5 (PMC5353951; doi:10.1186/s12889-017-4171-5)
Supplement: Additional file 1: Figure S1. — Children’s energy consumption in quick-service restaurants. (DOCX 18 kb) [file 12889_2017_4171_MOESM1_ESM.docx]

Additional file 1: Figure S1. Children’s energy consumption in quick-service restaurants
